# Hand-Selective Visual Regions Represent How to Grasp 3D Tools: Brain Decoding during Real Actions

**DOI:** 10.1523/JNEUROSCI.0083-21.2021

**Published:** 2021-06-16

**Authors:** Ethan Knights, Courtney Mansfield, Diana Tonin, Janak Saada, Fraser W. Smith, Stéphanie Rossit

**Affiliations:** ^1^Medical Research Council Cognition and Brain Sciences Unit, University of Cambridge, Cambridge CB2 7EF, United Kingdom; ^2^School of Psychology, University of East Anglia, Norwich NR4 7TJ, United Kingdom; ^3^Department of Radiology, Norfolk and Norwich University Hospitals NHS Foundation Trust, Norwich NR4 7UY, United Kingdom

**Keywords:** body representation, category selectivity, grasping, multivariate pattern analysis, tool use

## Abstract

Most neuroimaging experiments that investigate how tools and their actions are represented in the brain use visual paradigms where tools or hands are displayed as 2D images and no real movements are performed. These studies discovered selective visual responses in occipitotemporal and parietal cortices for viewing pictures of hands or tools, which are assumed to reflect action processing, but this has rarely been directly investigated. Here, we examined the responses of independently visually defined category-selective brain areas when participants grasped 3D tools (*N* = 20; 9 females). Using real-action fMRI and multivoxel pattern analysis, we found that grasp typicality representations (i.e., whether a tool is grasped appropriately for use) were decodable from hand-selective areas in occipitotemporal and parietal cortices, but not from tool-, object-, or body-selective areas, even if partially overlapping. Importantly, these effects were exclusive for actions with tools, but not for biomechanically matched actions with control nontools. In addition, grasp typicality decoding was significantly higher in hand than tool-selective parietal regions. Notably, grasp typicality representations were automatically evoked even when there was no requirement for tool use and participants were naive to object category (tool vs nontools). Finding a specificity for typical tool grasping in hand-selective, rather than tool-selective, regions challenges the long-standing assumption that activation for viewing tool images reflects sensorimotor processing linked to tool manipulation. Instead, our results show that typicality representations for tool grasping are automatically evoked in visual regions specialized for representing the human hand, the primary tool of the brain for interacting with the world.

## Significance Statement

The unique ability of humans to manufacture and use tools is unsurpassed across the animal kingdom, with tool use considered a defining feature of our species. Most neuroscientific studies that investigate the brain mechanisms that support tool use record brain activity while people simply view images of tools or hands and not when people perform actual hand movements with tools. Here we show that specific areas of the human visual system that preferentially process hands automatically encode how to appropriately grasp 3D tools, even when no actual tool use is required. These findings suggest that visual areas optimized for processing hands represent fundamental aspects of tool grasping in humans, such as which side they should be grasped for correct manipulation.

## Introduction

The emergence of handheld tools (e.g., a spoon) marks the beginning of a major discontinuity between humans and our closest primate relatives (Ambrose, 2001). Unlike other manipulable objects (e.g., books), tools are tightly associated with predictable motor routines (Johnson-Frey et al., 2003). A highly replicable functional imaging finding is that simply viewing tool pictures activates sensorimotor brain areas (Lewis, 2006), but what drives this functional selectivity? One popular idea is that this visually evoked activation reflects the automatic extraction of information about the actions that tools afford, like the hand movements required for their use (Martin et al., 1996; Fang and He, 2005). Similarly, tool-selective visual responses in supramarginal gyrus (SMG) or posterior middle temporal gyrus (pMTG) are often interpreted as indirect evidence that these regions are involved in real tool manipulation (Buxbaum et al., 2006; Bach et al., 2010). Nevertheless, we would never grasp a picture of a tool, and, more importantly, finding spatially overlapping activation between two tasks does not directly imply that the same neural representations are being triggered (Dinstein et al., 2008; Martin, 2016). In fact, intraparietal activation for viewing tool pictures versus grasping shows poor correspondence (Valyear et al., 2007; Gallivan et al., 2013), questioning the long-standing assumption that visual tool selectivity represents sensorimotor aspects of manipulation.

Curiously, the visual regions activated by viewing pictures of hands in the left intraparietal sulcus (IPS-hand) and lateral occipital temporal cortex (LOTC-hand) overlap with their respective tool-selective areas (IPS-tool; LOTC-tool; Bracci et al., 2012; Bracci and Peelen, 2013; Bracci and Op de Beeck, 2016). Stimulus features often described to drive the organization of category-selective areas, like form (Coggan et al., 2016), animacy (Konkle and Caramazza, 2013), or manipulability (Mahon et al., 2007), poorly explain this shared topography because hands and tools differ on these dimensions. Instead, their overlap is suggested to result from a joint representation of high-level action information related to skillful object manipulation (Bracci et al., 2012; Bracci and Op de Beeck, 2016; Striem-Amit et al., 2017), perhaps coding the function of hand configurations (Perini et al., 2014; Bracci et al., 2018). Arguably, the only way to directly test whether tool- or hand-selective visual areas carry information about tool actions is to examine their responses during real 3D tool manipulation. Yet, very few fMRI studies involve real tool manipulation (Gallivan et al., 2009; Valyear et al., 2012; Brandi et al., 2014; Styrkowiec et al., 2019). To date, only Gallivan et al. (2013) have investigated real tool manipulation in visually defined tool-selective regions and showed that IPS-tool/LOTC-tool are indeed sensitive to coarsely different biomechanical actions (reaching vs grasping) with a pair of tongs. However, it remains unknown whether hand-selective visual areas represent properties of real hand movements with 3D tools, like the way they are typically grasped for subsequent use.

**Figure 1.**
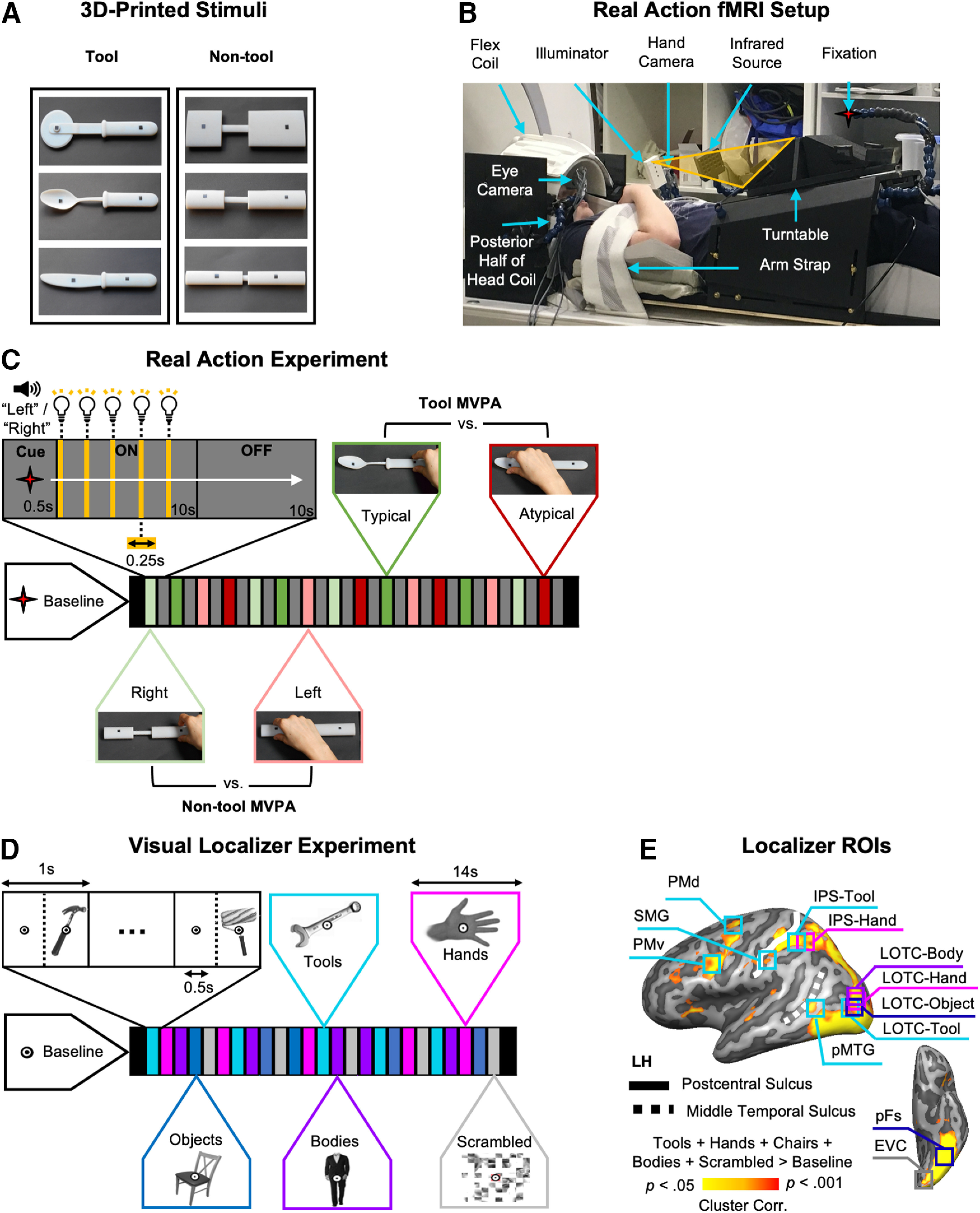
Experimental setup and design. ***A***, 3D-printed tool and nontool control object pairs (black markers on objects indicate grasp points), which were matched for elongation, width, and depth such that tool and nontool actions were biomechanically similar. ***B***, Side view of real-action participant setup used to present 3D objects at grasping distance (without the use of mirrors). Red star indicates fixation LED. The hand is shown at its starting position. ***C***, Timing and grasping tasks from the subject’s point of view for the real-action experiment. During the 10 s ON-block, the object was illuminated five times cueing the participant to grasp the object each time by its left or right side (as per preceding auditory cue) with the right hand. Exemplar videos of trial types can be accessed here: https://osf.io/gsmyw/. This was followed by a 10 s OFF-block involving no stimulation where the workspace remained dark. For MVPA, we treated tool and nontool trials independently, where for the tools only, right- and left-sided grasps were typical and atypical grasps, respectively (based on handle orientation). ***D***, Timing of visual localizer experiment. In the visual localizer, blocks of tools, hands, chairs, bodies, and scrambled 2D image stimuli were presented in between fixation-only screens. ***E***, For each individual participant, independent ROIs were defined for MVPA using functional activity from the visual localizer (Table 1). The representative ROI locations are displayed on a group activation contrast map from the visual localizer [all conditions > (Baseline*5)] projected onto a left hemisphere cortical surface reconstruction of a reference brain (COLIN27 Talairach) available from the neuroElf package (http://neuroelf.net).

Here, an fMRI experiment involving real hand actions (Fig. 1) tested whether visually defined hand- and tool-selective areas represented how to typically grasp 3D tools. Specifically, participants grasped 3D-printed tools in ways either consistent with their use (typical: by their handle) or not (atypical: by their functional end; e.g., knife blade). As a control, nontool bars (matched with the tools for elongation, width, and depth; Brandi et al., 2014) were also grasped on their right or left sides to match as much as possible any biomechanical differences between typical and atypical actions. Multivoxel pattern analysis (MVPA) was used to assess whether different tool grasps (typical vs atypical) and nontool grasps (right vs left) could be decoded from fMRI activity patterns within independent visually defined regions of interest (ROIs). Greater-than-chance decoding accuracy of typical versus atypical actions for tools, but not control nontools, was interpreted as evidence that an area contains high-level typicality representations about how a tool should be grasped correctly for use (i.e., by its handle). This pattern of findings was expected only for the tool- and hand-selective areas since these are thought to support tool manipulation (Mahon and Caramazza, 2009; Striem-Amit et al., 2017).

**Table 1:** Visual localizer ROI descriptives

ROI	Subjects with ROI (*N*)	Mean size (SD)	Mean peak coordinates (SD)		
			*x*	*y*	*z*
EVC	19	114 (35)	−14 (6)	−89 (4)	−9 (9)
LOTC-object	19	148 (34)	−42(4)	−77 (4)	−7 (4)
LOTC-body	18	55 (30)	−45 (3)	−76 (5)	2 (6)
LOTC-hand	17	81 (44)	−47 (4)	−71 (4)	−1 (5)
LOTC-tool	17	77 (45)	−47 (5)	−71 (5)	−2 (6)
pMTG	17	96(48)	−45 (4)	−57 (3)	3 (4)
pFs	19	105 (41)	−40 (4)	−54 (4)	−14 (4)
SMG	17	69 (43)	−53 (6)	−28 (4)	27 (6)
IPS-hand	19	110 (57)	−38 (4)	−46 (7)	42 (3)
IPS-tool	19	81 (55)	−37 (5)	−41 (7)	42 (5)
PMv	14	61 (42)	−45 (7)	−1 (6)	31 (5)
PMd	14	47 (28)	−29 (5)	−13 (4)	51 (4)

ROI subject counts with their mean sizes (voxels) and peak coordinates (Talairach).

## Materials and Methods

### 

#### Participants

Twenty healthy participants (11 males) completed the real-action fMRI experiment followed by a visual localizer experiment on a separate day. Data from one participant (male) were excluded from statistical analysis because of excessive head movements during the real-action experiment (i.e., translation and rotation exceeded 1.5 mm and 1.5° rotation), leaving a total sample of 19 participants (mean age, 23 ± 4.2 years; age range, 18–34 years). All participants had normal or corrected-to-normal vision and no history of neurologic or psychiatric disorders, were right handed (Oldfield, 1971), and gave written consent in line with procedures approved by the School of Psychology Ethics Committee at the University of East Anglia.

#### Real-action 3D stimuli

Tool and nontool object categories were designed (Autodesk) and 3D-printed (Objet30 Desktop) in VeroWhite material (Statasys): three common kitchen tools (knife, spoon, and pizzacutter) and three nontool control bars (Fig. 1*A*). Objects were secured to slots placed onto black pedestals used for stimulus presentation. Tools had identical handles (length × width × depth dimensions, 11.6 × 1.9 × 1.1 cm) with different functional ends attached (knife, 10.1 × 1.9 × 0.2 cm; spoon, 10.1 × 4.1 × 0.7 cm; pizzacutter, 10.1 × 7.5 × 0.2 cm). To avoid motor or visual confounds, tools and nontool pairs were carefully matched in terms of visual properties and kinematic requirements as much as possible. Specifically, nontools were composed of three cylindrical shapes (adapted from Brandi et al., 2014) with handle, neck, and functional end dimensions matched to each tool they controlled for, ensuring that grip size was matched between tool and nontool pairs. In addition, all objects had small black stickers placed at prespecified locations to indicate grasp points, ensuring that grasp position/reach distance were identical between tool and nontool pairs regardless of the side to be grasped. To avoid familiarity confounds between tools and nontool control stimuli, we chose to use bars instead of scrambled tools, and, thus, our control nontools were familiar, but had no specific associated function. Furthermore, each tool and nontool pair were carefully matched for elongation so that any differences between conditions could not be explained by low-level shape preferences (Sakuraba et al., 2012; Brandi et al., 2014).

#### Real-action setup and apparatus

Participants were scanned in complete darkness using a head-tilted configuration that allowed direct viewing of the workspace and 3D stimuli without the use of mirrors (Fig. 1*B*) by tilting the head coil ∼20° and padding the underside of each participants heads with foam cushions (NoMoCo Pillow). Objects were placed by an experimenter on a turntable above the participant’s pelvis and were visible only when illuminated (Fernández-Espejo et al., 2015; Fig. 1*B*). All stimuli were mounted such that they were aligned with participants’ midlines, never changed position while visible and were tilted away from the horizontal at an angle (∼15°) to maximize visibility and grasp comfort. For stimulus presentation, the workspace and object were illuminated from the front using a bright white light-emitting diode (LED) attached to a flexible plastic stalk (Loc-line, Lockwood Products; Fig. 1*B*). To control for eye movements, participants were instructed to fixate a small red LED positioned above and behind objects such that they appeared in the lower visual field (Rossit et al., 2013). Throughout the experiment, a participant’s right eye and arm movements were monitored online and recorded using two MR-compatible infrared-sensitive cameras (MRC Systems) to verify that participants performed the correct grasping movement (hand camera positioned over the left shoulder; Fig. 1*B*) and maintained fixation (eye camera beside the right eye; Fig. 1*B*). The likelihood of motion artifacts related to grasping was reduced by restraining the upper right arm and providing support with additional cushions so that movements were performed by flexion around the elbow only (Culham, 2006). Auditory instructions were delivered to the participants through earphones (MRI-Compatible Insert Earphones, Model S14, SENSIMETRICS). At the beginning of the real-action session, participant setup involved adjusting the exact position of the following: (1) stimuli and the hand to ensure reachability (average grasping distance between the “home” position and object, 43 cm); (2) the illuminator to equally light all objects; (3) the fixation LED to meet the natural line of gaze (average distance from fixation to bridge nose, 91 cm; visual angle, ∼20°); and (4) the infrared-sensitive eye and hand cameras to monitor eye and hand movement errors. The experiment was controlled by a MATLAB script (version R2010a; MathWorks) using the Psychophysics Toolbox (Brainard, 1997).

#### Real-action experimental paradigm

We used a powerful block design fMRI paradigm, which maximized the contrast-to-noise ratio to generate a reliable estimate of the average response pattern (Mur et al., 2009) and improved detection of blood oxygenation level-dependent (BOLD) signal changes without significant interference from artifacts during overt movement (Birn et al., 2004). A block began with an auditory instruction (“left” or “right”; 0.5 s) specifying which side of the upcoming object to grasp (Fig. 1*C*). During the ON-block (10 s), the object was briefly illuminated for 0.25 s five consecutive times (within 2 s intervals) cueing the participant to grasp with a right-handed precision grip (i.e., index finger and thumb) along the vertical axis. Between actions, participants returned their hand to a “home” position with their right hand closed in a fist on their chest (Fig. 1*B*). This brief object flashing presentation cycle during ON-blocks has been shown to maximize the signal-to-noise ratio in previous perceptual decoding experiments (Kay et al., 2008; Smith and Muckli, 2010) and eliminates the sensory confound from viewing hand movements (Rossit et al., 2013; Monaco et al., 2015). An OFF-block (10 s) followed the stimulation block where the workspace remained dark and the experimenter placed the next stimulus. A single fMRI run included 16 blocks involving the four grasping conditions (i.e., typical tool, atypical tool, right nontool, and left nontool) each with three repetitions (one per exemplar; every object was presented twice and grasped on each side once). An additional tool (whisk) and a nontool object were presented on the remaining four blocks per run, but not analyzed as they were not matched in dimensions because of a technical problem (the original control nontool for the whisk was too large to allow rotation of the turntable within the scanner bore). On average, participants completed six runs (minimum, five runs; maximum, seven runs) for a total of 18 repetitions per grasping condition. Block orders were pseudorandomized such that conditions were never repeated (two-back) and were preceded an equal number of times by other conditions. Each functional scan lasted 356 s, inclusive of start/end baseline fixation periods (14 s). Each experimental session lasted ∼2.25 h (including setup, task practice, and anatomic scan). Before the fMRI experiment, participants were familiarized with the setup and practiced the grasping tasks in a separate laboratory session (30 min) outside of the scanner. The hand and eye movement videos were monitored online and offline to identify error trials. Two runs (of two separate participants) from the entire dataset were excluded from further analysis. In one of these blocks, the participant failed to follow the grasping task instructions correctly (i.e., performing alternated left and right grasps) and for the remaining block another participant did not maintain fixation (i.e., made downward saccades toward objects). In the remaining runs that were analyzed, participants made performance errors in <1% of experimental trials. The types of errors included the following: not reaching (three trials, two participants), reaching in the wrong direction (one trial, one participant), and downward eye saccades (five trials, three participants). A one-way repeated-measures ANOVA with 12 levels (i.e., the six exemplars across both left vs right grasping conditions) showed that the percentage of errors was equally distributed among trial types regardless of whether the percentage of hand and eye errors were combined or treated separately (all *p* values > 0.28).

Crucially, since the tool handles were always oriented rightward, the right and left tool trials involved grasping tools either by their handle (typical) or functional end (atypical), respectively. On the other hand, grasping nontools did not involve a typical manipulation but only differed in grasp direction with right versus left grasps (Fig. 1*C*). We chose to present rightward-oriented tool handles only, rather than alternate object orientation randomly between trials, to reduce total trial numbers (scanning times were already quite extensive with setup) and because of technical limitations (i.e., the rotation direction of the turntable was fixed, and it was difficult for the experimenter to manipulate tool orientation in the dark). Nevertheless, by comparing the decoding accuracies for each region between tool and nontool grasps (which were matched for biomechanics), we ruled out the possibility that our typical manipulation simply reflected grasp direction. Specifically, we took the conservative approach that for an area to be sensitive to tool grasping typicality, it should not only show greater-than-chance decoding for typical versus atypical actions with tools (i.e., typicality), but also that the typicality decoding accuracy should be significantly greater than the accuracy for biomechanically matched actions with our control nontools (i.e., right vs left actions with nontools).

#### Visual localizer

On a separate day from the real-action experiment, participants completed a bodies, chairs, tools and hands (BOTH) visual localizer (adapted from Bracci et al., 2012; Bracci and Peelen, 2013; Bracci and Op de Beeck, 2016) using a standard coil configuration (for details, see MRI acquisition). Two sets of exemplar images were selected from previous stimuli databases (Bracci et al., 2012; Bracci and Peelen, 2013; Bracci and Op de Beeck, 2016) that were chosen to match, as much as possible, the characteristics within the tool (i.e., identity and orientation), body (i.e., gender, body position, and amount of skin shown), hand (i.e., position and orientation), and chair (i.e., materials, type, and style) categories. Using a mirror attached to the head coil, participants viewed separate blocks (14 s) of 14 different grayscale 2D pictures from a given category (400 × 400 pixels; 0.5 s). Blank intervals separated individual stimuli (0.5 s), and scrambled image blocks separated cycles of the four randomized category blocks (Fig. 1*D*). Throughout, participants fixated on a superimposed bullseye in the center of each image and, to encourage attention, performed a one-back repetition detection task where they made a right-handed button press whenever two successive photographs were identical. The 2D image stimuli were presented with an LCD projector (SilentVision SV-6011 LCD, Avotech). A single fMRI run included 24 category blocks (six repetitions per condition) with blank fixation baseline periods (14 s) at the beginning and the end of the experiment. Each localizer scan lasted 448 s, and, on average, participants completed four runs (minimum, three runs; maximum, four runs) for a total of 24 repetitions/condition. The entire localizer session lasted ∼50 min after including the time taken to acquire a high-resolution anatomic scan and to set up participants.

#### MRI acquisition

The BOLD fMRI measurements were acquired using a 3 T wide-bore MR scanner (Discovery MR750, GE Healthcare) at the Norfolk and Norwich University Hospital (Norwich, UK). To achieve a good signal-to-noise ratio during the real-action fMRI experiment, the posterior half of a 21-channel receive-only coil was tilted and a 16-channel receive-only flex coil was suspended over the anterosuperior part of the skull (Fig. 1*B*). A T2*-weighted single-shot gradient echoplanar imaging sequence was used throughout the real-action experiment to acquire 178 functional MRI volumes [repetition time (TR) = 2000 ms; voxel resolution (VR) = 3.3 × 3.3 × 3.3 mm; echo time (TE) = 30 ms; flip angle (FA) = 78°; field of view (FOV) = 211 × 211 mm; matrix size (MS) = 64 × 64], which comprised 35 oblique slices (no gap) acquired at 30° with respect to anterior commissure–posterior commissure (AC–PC), to provide near whole-brain coverage. A T1-weighted anatomic image with 196 slices was acquired at the beginning of the session using BRAVO sequences (TR = 2000 ms; TE = 30 ms; FOV = 230 × 230 × 230 mm; FA = 9°; MS = 256 × 256; voxel size = 0.9 × 0.9 × 0.9 mm).

For visual localizer sessions, a full 21-channel head coil was used to obtain 224 functional MRI volumes (TR = 2000 ms; VR = 3.3 × 3.3 × 3.3 mm; TE = 30 ms; FA = 78°; FOV = 211 × 211 mm; MS = 64 × 64). A high-resolution T1-weighted anatomic image with 196 slices was acquired before the localizer runs (TR = 2000 ms; TE = 30 ms; FOV = 230 × 230 × 230 mm; FA = 9°; MS = 256 × 256; voxel size = 0.9 × 0.9 × 0.9 mm). Localizer datasets for two participants were retrieved from another study from our group (Rossit et al., 2018), where the identical paradigm was performed when acquiring volumes using a whole-body 3 T scanner (MAGNETOM Prisma Fit, Siemens) with a 64-channel head coil and integrated parallel imaging techniques at the Scannexus imaging center (Maastricht, The Netherlands) and comparable acquisition parameters (functional scans: TR = 2000 ms; TE = 30 ms; FA = 77°; FOV = 216 mm; MS = 72 × 72; anatomical scan, T1-weighted anatomic image: TR = 2250 ms; TE = 2.21 ms; FA = 9°; FOV = 256 mm; MS = 256 × 256).

#### Data preprocessing

Preprocessing and ROI definitions were performed using BrainVoyager QX (version 2.8.2; Brain Innovation). The BrainVoyager 3D motion correction (sinc interpolation) aligned each functional volume within a run to the functional volume acquired closest in time to the anatomic scan (Rossit et al., 2013). Slice scan time correction (ascending and interleaved) and high-pass temporal filtering (two cycles per run) was also performed. Functional data were superimposed on to the anatomic brain images acquired during the localizer paradigm that were previously aligned to the plane of the AC–PC and transformed into standard stereotaxic space (Talairach and Tournoux, 1988). Excessive motion was screened by examining the time-course movies and motion plots created with the motion-correction algorithms for each run. No spatial smoothing was applied.

To estimate activity in the localizer experiment, a predictor was used per image condition (i.e., bodies, objects, tools, hands, and scrambled) in a single-subject general linear model. Predictors were created from boxcar functions that were convolved with a standard 2*y* model of the hemodynamic response function (Boynton et al., 1996) and aligned to the onset of the stimulus with durations matching block length. The baseline epochs were excluded from the model, and therefore, all regression coefficients were defined relative to this baseline activity. This process was repeated for the real-action experiment, using 16 separate predictors for each block of stimulation independently per run (12 exemplars, e.g., knife typical, knife atypical, spoon typical, plus four foil trials) and 6 motion regressors (confound predictors). These estimates (β weights) from the real-action experiment were used as the input to the pattern classifier.

#### Visual localizer regions of interest

Twelve visual ROIs were defined at the individual participant level from the independent BOTH localizer data by drawing a cube (15 voxels^3^) around the peak of activity from previously reported volumetric contrasts (see below; Fig. 1*E*, Table 1) set at a threshold of *p *<* *0.005 (Gallivan et al., 2013) or, if no activity was identified, of *p *<* *0.01 (Bracci and Op de Beeck, 2016). In cases where no activity was observed, the ROI was omitted for that participant (Table 1). Given the predominantly left lateralized nature of tool processing (Lewis, 2006); all individual participant ROIs were defined in the left hemisphere (Bracci et al., 2012; Bracci and Peelen, 2013; Peelen et al., 2013; Bracci and Op de Beeck, 2016). Six tool-selective ROIs commonly described in left frontoparietal and occipitotemporal cortices were identified by contrasting activation for tool pictures versus other object pictures [IPS-tool; SMG; dorsal premotor cortex (PMd); ventral premotor cortex (PMv), LOTC-tool; pMTG; Martin et al., 1996; Grafton et al., 1997]. Moreover, two hand-selective ROIs were identified in LOTC (LOTC-hand) and IPS (IPS-hand) by contrasting activation for hand pictures versus pictures of other body parts (Bracci et al., 2012, 2018; Peelen et al., 2013; Bracci and Op de Beeck, 2016; Palser and Cavina-Pratesi, 2018). Additionally, we defined a body-selective ROI (LOTC-body; bodies > chairs; Bracci and Op de Beeck, 2016), two object-selective ROIs [LOTC-object selective (LOTC-object); posterior fusiform (pFs); chairs > scrambled; Bracci and Op de Beeck, 2016; Hutchison et al., 2014], and an early visual cortex ROI (EVC; all categories > baseline; Bracci and Op de Beeck, 2016). The ROI locations were verified by a senior author (S.R.) with respect to the following anatomic guidelines and contrasts.

##### LOTC-object.

LOTC-object (chairs > scrambled; Hutchison et al., 2014; Bracci and Op de Beeck, 2016) is defined by selecting the peak of activation near the lateral occipital sulcus (LOS; Hutchison et al., 2014; Bracci and Op de Beeck, 2016; Malach et al., 1995; Grill-Spector et al., 1999, 2001).

##### LOTC-body.

LOTC-body (bodies > chairs; Bracci and Op de Beeck, 2016) is defined by selecting the peak of activation near the LOS and inferior to the left extrastriate body area (EBA; Valyear and Culham, 2010), which was identified by the contrast [(bodies + hands) > chairs; adapted from Bracci et al., 2010; (whole bodies + body parts) > (hands + chairs)]. EBA was not included in the analysis.

##### LOTC-hand.

LOTC-hand [(hands > Chairs) and (hands > bodies); Bracci and Op de Beeck, 2016] is defined by selecting the peak of activation near the LOS. These were often anterior to LOTC-body (Bracci et al., 2010; Bracci and Op de Beeck, 2016).

##### LOTC-tool.

LOTC-tool (tools > chairs; Bracci et al., 2012; Hutchison et al., 2014) is defined by selecting the peak of activation near the LOS. These often closely overlapped LOTC-hand (Bracci et al., 2012).

##### pMTG.

pMTG (tools > chairs; Hutchison et al., 2014; Valyear and Culham, 2010) is defined by selecting the peak of activation on the pMTG, more lateral, ventral, and anterior to EBA (Hutchison et al., 2014). We selected the peak anterior to the anterior occipital sulcus (AOS), as the MTG is in the temporal lobe and the AOS separates the temporal lobe from the occipital lobe (Damasio, 1995).

##### pFs.

pFs (chairs > scrambled; Hutchison et al., 2014) is defined by selecting the peak of activation in the posterior aspect of the fusiform gyrus, extending into the occipitotemporal sulcus (Hutchison et al., 2014).

##### IPS-hand.

IPS-hand (hands > chairs; Bracci and Op de Beeck, 2016) is defined by selecting the peak of activation on the IPS (Bracci and Op de Beeck, 2016).

##### IPS-tool.

IPS-tool (tools > scrambled; Bracci and Op de Beeck, 2016) is defined by selecting the peak of activation on the IPS (Bracci and Op de Beeck, 2016).

##### SMG.

SMG (tools > scrambled; Creem-Regehr et al., 2007) is defined by selecting the peak of activation located most anterior along the SMG (Peeters et al., 2013), lateral to the anterior segment of the IPS (Gallivan et al., 2013), posterior to the precentral sulcus (PreCS), and superior to the lateral sulcus (Ariani et al., 2015).

##### PMd.

PMd (tools > scrambled) is defined by selecting the peak of activation at the junction of the PreCS and the superior frontal sulcus (Gallivan et al., 2013; Ariani et al., 2015).

##### PMv.

PMv (tools > scrambled; Creem-Regehr et al., 2007) is defined by selecting the voxels inferior and posterior to the junction between the inferior frontal sulcus and the PreCS (Gallivan et al., 2013).

##### EVC.

EVC (all conditions > baseline; Bracci and Op de Beeck, 2016) is defined by selecting the voxels in the occipital cortex near the calcarine sulcus (Singhal et al., 2013).

#### Pattern classification

We performed MVPA independently for tool and nontool trial types. Independent linear pattern classifiers [linear support vector machine (SVM)] were trained to learn the mapping between a set of brain-activity patterns (β values computed from single blocks of activity) from the visual ROIs and the type of grasp being performed with the tools (typical vs atypical) or nontools (right vs left). To test the performance of our classifiers, decoding accuracy was assessed using an *n*-fold leave-one-run-out cross-validation procedure; thus, our models were built from *n* – 1 runs and were tested on the independent *n*th run (repeated for the *n* different possible partitions of runs in this scheme; Duda et al., 2001; Smith and Muckli, 2010; Smith and Goodale, 2015; Gallivan et al., 2016) before averaging across *n* iterations to produce a representative decoding accuracy measure per participant and per ROI. Beta estimates for each voxel were normalized (separately for training and test data) within a range of −1 to +1 before input to the SVM (Chang and Lin, 2011), and the linear SVM algorithm was implemented using the default parameters provided in the LibSVM toolbox (*C* = 1). Pattern classification was performed with a combination of in-house scripts (Smith and Muckli, 2010; Smith and Goodale, 2015) using MATLAB with the Neuroelf toolbox (version 0.9c; http://neuroelf.net) and a linear SVM classifier (libSVM 2.12 toolbox; https://www.csie.ntu.edu.tw/∼cjlin/libsvm/).

#### Statistical analysis

**Figure 2.**
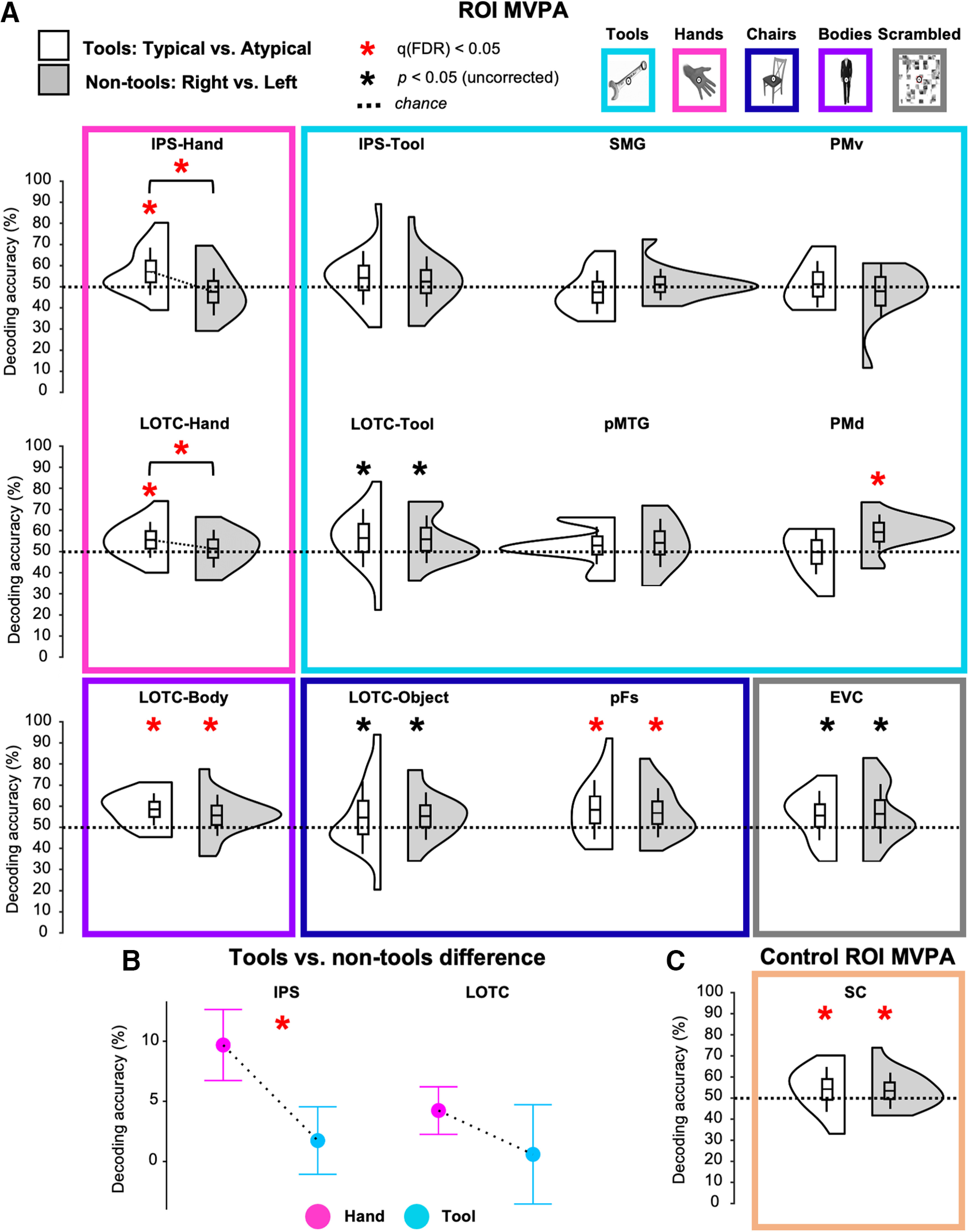
Grasp type decoding results in left hemisphere ROIs. ***A***, Violin plots of MVPA data from visual localizer ROIs for the typical versus atypical classification of grasping tools (white violins) and nontool control grasping (right vs left decoding; gray violins). Box plot center lines are the mean decoding accuracy, while their edges and whiskers show ±1 SD and ±2 SEMs, respectively. Decoding accuracies of typical versus atypical grasping in IPS and LOTC hand-selective cortex (pink) are significantly greater than chance for tools, but not for nontools. ***B***, ANOVA results comparing the difference of decoding accuracy between tools (typical vs atypical) and nontools (right vs left) for the partially overlapping hand- and tool-selective ROIs within the IPS and LOTC. ***C***, Violin plot of MVPA data for control ROI in SC based on an independent contrast (all actions > baseline) from real-action experiment showing significant decoding of grasp type for both tools and nontools. Red asterisks show FDR-corrected results, while black asterisks show uncorrected results.

One-tailed one-sample *t* tests were used to test for above-chance decoding for tool and nontool action classifications in every ROI independently. If the pattern of results was consistent with our hypothesis (i.e., decoding accuracy was significantly above chance for tools, but not for nontools), we further ran a one-tailed pairwise *t* test to compare whether decoding accuracy was significantly higher for tools than for nontools. Additionally, to test for differences in decoding accuracy between ROIs, we used repeated-measures 2 × 2 ANOVAs with ROI (tool vs hand selective) and region (IPS vs. LOTC) as within-subject factors. Then, to test whether univariate differences would differ between grasp types for the tools, but not for the nontools, we ran 2 × 2 ANOVAs with grasp type (typical/right vs atypical/left) and object category (tools vs nontools) by entering mean β weights for each ROI. Separately for each set of analyses, we corrected for multiple comparisons with false discovery rate (FDR) correction of *q* ≤ 0.05 (Benjamini and Hochberg, 1995; Benjamini and Yekutieli, 2001) across the number of tests. Only significant results are reported (Fig. 2). Our sample size was based on similar motor studies using MVPA (Gallivan et al., 2009, 2013, 2014; Ariani et al., 2015, 2018), though no power analysis was performed before data collection.

To test for evidence for the null hypothesis over an alternative hypothesis, we supplemented null-hypothesis significance tests with Bayes factors (BFs; Wagenmakers, 2007; Rouder et al., 2009). Bayes factors were estimated using the bayesFactor toolbox in MATLAB (version 1.1; https://klabhub.github.io/bayesFactor). The Jeffreys–Zellner–Siow default prior on effect sizes was used (Rouder et al., 2012), and BFs were interpreted according to the criteria set out by Jeffreys (1961; Jarosz and Wiley, 2014), where a BF_01_ between 1 to 3 and > 3 indicate “anecdotal” and “substantial” evidence in favor of the null, respectively.

#### Data availability

Stimuli, code for running the experiment and for MVPA analyses, and ROI data are accessible from Open Science Framework at https://osf.io/zxnpv. Full raw MRI dataset (real action and visual localizer) is accessible from OpenNEURO at https://openneuro.org/datasets/ds003342/versions/1.0.0.

## Results

In line with our predictions, as can be seen in Figure 2, a one-sample *t* test against chance (50%) showed that SVM decoding accuracy (FDR corrected) from hand-selective ROIs in LOTC and IPS were significantly greater-than-chance when discriminating typical versus atypical actions with tools (mean ± SD; LOTC-hand accuracy = 56 ± 0.9%, *t*_(16)_ = 2.73, *p *=* *0.007, *d *=* *0.66; IPS-hand accuracy = 57 ± 0.11%, *t*_(18)_ = 2.72, *p *=* *0.007, *d *=* *0.62), but not biomechanically matched actions with nontools (right vs left; LOTC-hand: *p *=* *0.252, IPS-hand: *p *=* *0.844). In fact, there was substantial evidence in favor of null decoding of nontool actions for the IPS ROI (LOTC-hand, BF_01_ = 2.29; IPS-hand, BF_01_ = 8.4). Importantly, results from a stringent between-classification paired-samples *t* test also further supported this: typicality decoding accuracy from both LOTC-hand and IPS-hand was significantly higher for tools than for biomechanically matched actions with nontools (LOTC-hand: *t*_(16)_ = 2.11, *p *=* *0.026, *d *=* *0.51; IPS-hand: *t*_(18)_ = 3.26, *p *=* *0.002, *d *=* *0.75; Fig. 2*A*,*B*).

No other visual ROI, including tool-selective areas, displayed the same significant effects as hand-selective areas (Fig. 2*A*,*B*). For tool-selective ROIs, decoding accuracy was not significantly greater than chance for classifying actions with tools or nontools (all *p* values > 0.024), with the Bayesian approach demonstrating strong evidence in favor of the null for PMv tool decoding (tool, BF_01_ = 3.23; nontool, BF_01_ = 6.85) and SMG tool decoding (tool, BF_01_ = 8.85; other BF_01_ values < 1.08). The exception to this was tool-selective PMd, which was found to decode significantly above-chance actions with nontools (accuracy = 59 ± 0.08%, *t*_(13)_ = 4.11, *p *=* *0.001, *d *=* *1.1; Fig. 2*A*), but not tools (BF_01_ = 4.42). As for object- and body-selective areas, LOTC-object decoding accuracy did not differ from chance for tools or nontools (*p *>* *0.026), though evidence in favor of the null was anecdotal (BF_01_ values < 1.33), whereas pFs and LOTC-body decoded actions above chance with both tools (pFs: accuracy = 58 ± 0.14%, *t*_(18)_ = 2.57, *p *=* *0.01, *d *=* *0.59; LOTC-body: accuracy = 59 ± 0.08%, *t*_(17)_ = 4.75, *p *<* *0.001, *d *=* *1.12) and nontools (pFs: accuracy = 57 ± 0.12%, *t*_(18)_ = 2.59, *p *=* *0.009, *d *=* *0.59; LOTC-body: accuracy = 56 ± 0.10%, *t*_(17)_ = 2.46, *p *=* *0.012, *d *=* *0.58; Fig. 2*A*). Like many of the tool-selective ROIs, the control EVC ROI was not found to decode actions with either type of object (*p* values < 0.026), albeit evidence in favor of the null was anecdotal (BF_01_ values < 0.43).

Since we obtained a different pattern of results for LOTC and IPS ROIs that were hand versus tool selective, we compared the decoding accuracies between these regions with a repeated-measures ANOVA with ROI (hands vs tool selective) and object category (tool vs nontools) as within factors. As shown in Figure 2*B*, there was a significant interaction between ROI and object category in IPS (*F*_(1,18)_ = 5.94, *p *=* *0.025, η^2^ = 0.25). *Post hoc t* tests showed that for IPS-hand, grasp-type decoding was significantly higher for tools than nontools (mean difference = 0.1%, SE = 0.03%; *p *=* *0.004), but not for IPS-tool (mean difference = 0.02%, SE = 0.03%). However, for LOTC this interaction was not significant (*p *=* *0.379; Fig. 2*B*), nor were the remaining main effects (all *p* values > 0.367).

**Figure 3.**
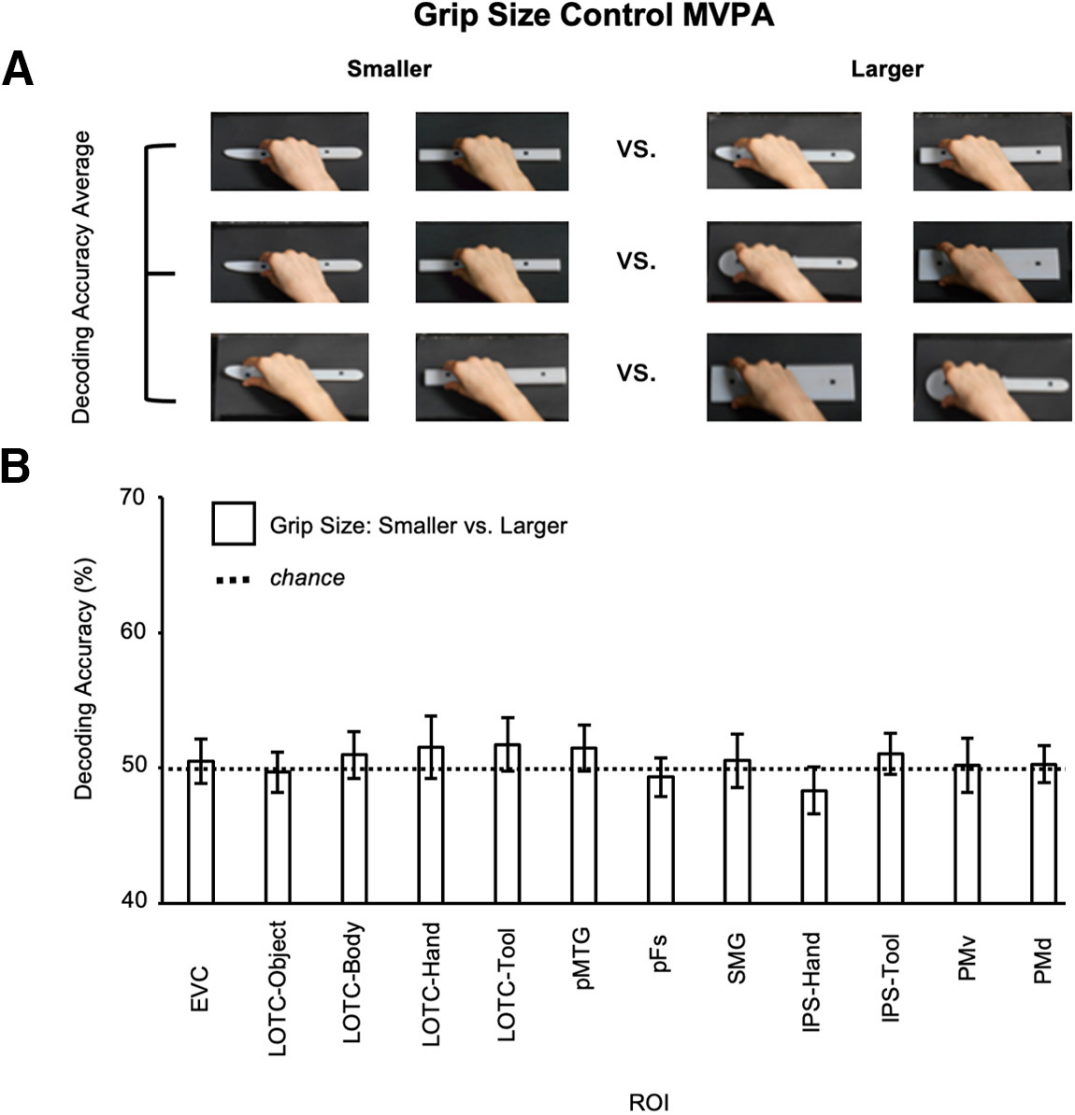
Grip size decoding. ***A***, We decoded smaller versus larger objects in three separate decoding analyses, regardless of whether the objects were tools or nontools. Each separate grip size pair decoding analysis is shown in each row of images in ***A*** (from top to bottom: small vs medium; small vs large; medium vs large). The heads of the knife, spoon, and pizzacutter tools and their paired nontools had matched small, medium, and large widths, respectively. Decoding accuracies for each grip size pair were then averaged and tested against chance using a one-tailed one-sample *t* test. In all cases, object category was collapsed to maximize power and generalizability (i.e., grasping tools and nontools), and reach direction was matched to minimize kinematic variance (i.e., all actions were leftward). ***B***, Mean decoding accuracy in visual localizer ROIs for the small versus large classification collapsed across object category. Error bars represent ±1 SEM.

Next, we examined whether significant decoding in hand-selective cortex could be accounted for by low-level sensory differences between the handles and functional ends of the tools. First, to test the possibility that tool-specific decoding in hand-selective cortex could be driven by simple textural differences (e.g., a smooth handle vs a serrated knife blade), we repeated the analysis using a left somatosensory cortex (SC) ROI defined by selecting the peak voxel in the postcentral gyrus in the same subjects with an independent univariate contrast of all grasps > baseline (Fabbri et al., 2014, 2016). However, unlike the higher accuracies for grasping tools than nontools in the hand-selective ROIs, grasp-type decoding in SC was significantly greater than chance for both tool (accuracy = 57 ± 0.11%, *t*_(18)_ = 3.04, *p *=* *0.004, *d *=* *0.7) and nontools (accuracy = 57 ± 0.09%, *t*_(18)_ = 3.45, *p *=* *0.001, *d *=* *0.79; Fig. 2*C*). This indicates that tool-specific decoding in hand-selective cortex cannot be solely explained by somatosensory differences in the stimuli. Second, we tested whether size differences between our objects, and thus grip size, could drive tool-specific decoding in hand-selective cortex (i.e., the functional end of the tool being wider than its handle for the spoon and pizza cutter). As shown in Figure 3*A*, we decoded smaller versus larger objects in three separate decoding analyses, regardless of whether the objects were tools or nontools. Each separate grip size pair decoding analysis is shown in each row of images of Figure 3*A* (from top to bottom: small vs medium; small vs large; medium vs large). Decoding accuracies for each grip size pair were then averaged and tested against chance using a one-tailed one-sample *t* test. Decoding of grip size was not significant for any visual ROI (all *p* values ≥ 0.1; Fig. 3*B*) and evidence in favor of the null was strong for most ROIs, including IPS-hand (BF_01_ = 8), EVC (BF_01_ = 3.22), LOTC-object (BF_01_ = 4.93), pFs (BF_01_ = 5.97), SMG (BF_01_ = 3.33), PMv (BF_01_ = 3.91), and PMd (BF_01_ = 3.56; all other BF_01_ values > 1.84). Together, these findings suggest that hand-selective regions, particularly in the IPS, are sensitive to whether a tool is grasped correctly by its handle or not, and that these effects are not simply because of textural or size differences between the stimuli used or actions performed.

**Figure 4.**
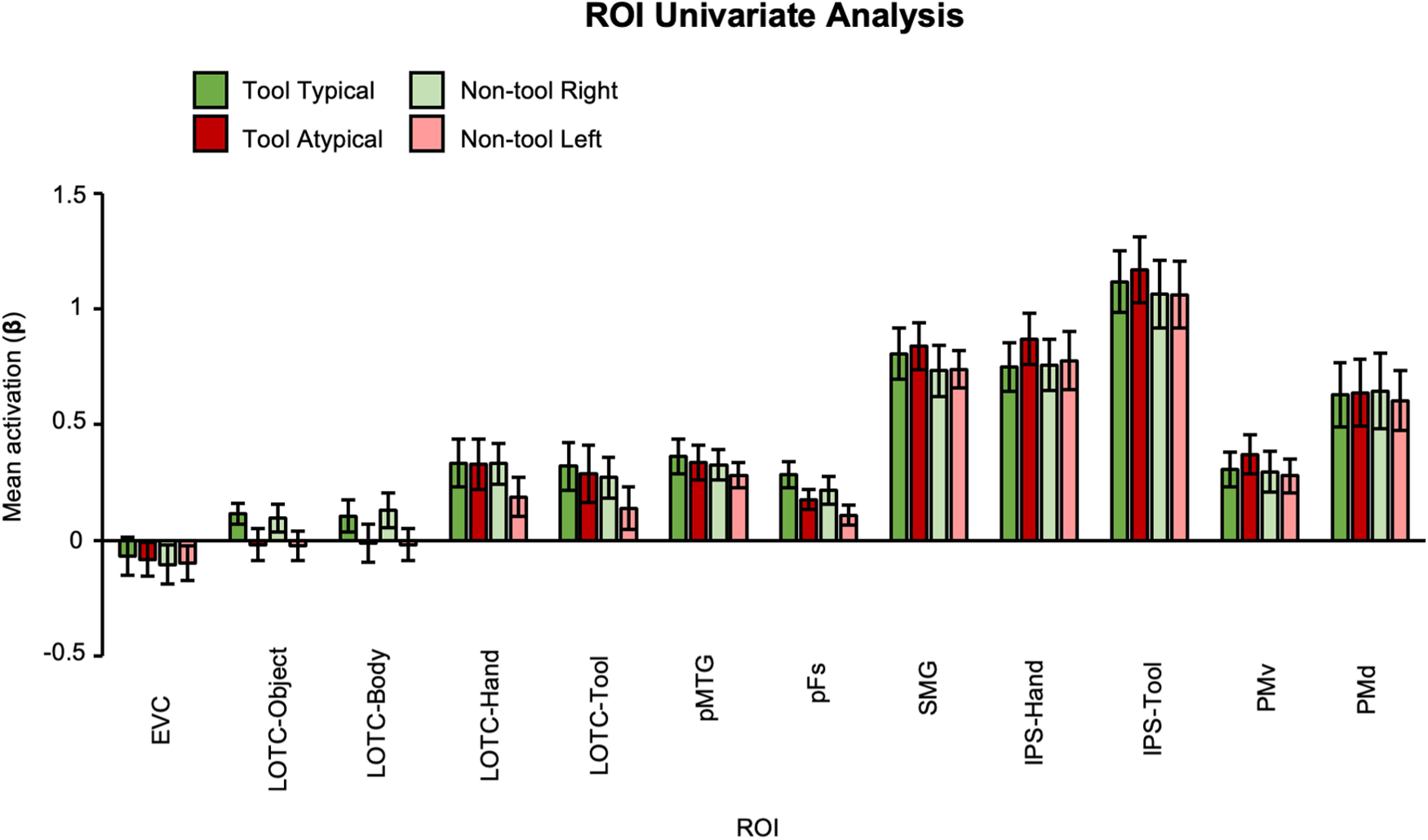
Mean activation (β) per ROI and condition used as input for pattern classification and univariate analyses. Error bars represent ±1 SEM.

In addition, we found that the significant decoding accuracies reported here do not simply reflect the overall response amplitudes within each ROI. When we analyzed the mean β weights in ANOVAs with grasp type and object category as within-subject factors for each ROI (i.e., as done in conventional univariate analysis; Fig. 4), the only significant effect observed was a main effect of object category (unrelated to typicality), where greater activation was found for tools relative to nontools in LOTC-tool (*F*_(1,16)_ = 9.25, *p *=* *0.008, η^2^ = 0.37; mean difference = 0.1, SE = 0.03), pFs (*F*_(1,18)_ = 8.68, *p *=* *0.009, η^2^ = 0.33; mean difference = 0.07, SE =  0.02), and SMG (*F*_(1,16)_ = 10.5, *p *=* *0.005, η^2^ = 0.4; mean difference = 0.089, SE = 0.03).

## Discussion

Our understanding of how the human brain represents object properties (Kanwisher, 2010) and simple hand movements (Gallivan and Culham, 2015) has significantly advanced in the last few decades; however, far less is known about the neural representations that underpin real actions involving 3D tools (Valyear et al., 2017). Most neuroimaging experiments that investigate how tools and their associated actions are represented in the brain have used visual paradigms where objects and body parts are displayed as 2D images (Ishibashi et al., 2016). These studies have discovered a tight anatomic and functional relationship between hand- and tool-selective areas in LOTC and IPS, thought to reflect action-related processing; however, this was yet to be directly tested (Bracci et al., 2012; Bracci and Peelen, 2013; Peelen et al., 2013; Bracci and Op de Beeck, 2016; Striem-Amit et al., 2017; Maimon-Mor and Makin, 2020). Here we defined visually category-selective areas and investigated whether they were sensitive to real-action affordances involving 3D tools. We found the first evidence that hand-selective cortex (left IPS-hand and LOTC-hand) represents whether a 3D tool is being grasped appropriately by its handle. Remarkably, the same effects were not observed in tool-, object-, or body-selective areas, even when these areas overlapped with hand-selective voxels in IPS and LOTC.

Our results indicate that visual hand-selective areas in parietal and occipital cortices process sensorimotor affordances of typicality for hand movements with 3D tools. Importantly, these action-related representations were detected exclusively for actions with tools, but not for biomechanically matched actions with nontools. This tool specificity was particularly evident in IPS-hand because Bayesian evidence demonstrated that the decoding of grasp type with nontools was not possible. In a similar vein, while the IPS ANOVA demonstrated boosted tool-specific decoding specifically for the hand-selective ROI, this effect was not significant in LOTC. This suggests that typicality effects may be less robust for LOTC-hand. Our findings shed light into the features of sensorimotor processing in hand-selective areas. First, their representations are sensitive to concepts acquired through experience (i.e., knowing how to grasp tools appropriately is a learned skill; Martin, 2007), fitting with evidence showing that learning about how to manipulate tools (Weisberg et al., 2007) or even performing such actions (Valyear et al., 2012; Brandi et al., 2014; Styrkowiec et al., 2019) affects LOTC and IPS activity. For example, our results are compatible with those from Brandi et al. (2014), who showed coactivation of these regions during “use” actions of tools/nontools. Our results, however, additionally suggest that this learned information, at least for grasping, is coded in specific category-selective parts of LOTC and IPS. Second, information processed by hand-selective cortex is represented in an abstract format beyond low-level properties (e.g., basic kinematics), since Bayesian evidence strongly suggested that decoding grip size was not possible. This fits well with reports that hand-/tool-selective overlap exists in people born without vision (Peelen et al., 2013) or without hands (Striem-Amit et al., 2017), suggesting that their development is driven by similarities in how they process nonsensory tool information. In addition, our data also resonate with previous studies showing that tool-selective areas in pMTG/LOTC and IPS represent abstract action goals (reach vs grasp) regardless of biomechanics (Jacobs et al., 2010; Gallivan et al., 2013), abeit our findings were observed for hand-selective areas only. Third, our study shows that these high-level representations are automatically evoked (Valyear et al., 2012) as throughout the real-action fMRI task there was no explicit requirement to use the tools and participants were never told that we were investigating “tools.” Here we demonstrate that these principles, frequently described to support tool use (Gibson, 1979; Imamizu et al., 2003; Maravita and Iriki, 2004; Umilta et al., 2008; Lingnau and Downing, 2015), apply to brain areas specialized for representing the human hand, our primary tool for interacting with the world.

An intriguing aspect of our results is that typicality decoding was successful using activity patterns from hand-selective cortex, but not overlapping parts of tool-selective cortex, in the LOTC and IPS. Bayesian evidence only anecdotally supported the possibility that decoding was null from tool-selective areas, but significantly stronger typicality decoding was observed for IPS-hand than IPS-tool during tool, but not nontool grasps. In contrast to previous picture-viewing fMRI studies showing that overlapping hand- and tool-selective regions exhibit similar responses (Bracci et al., 2012; Bracci and Peelen, 2013; Bracci and Op de Beeck, 2016), our findings uniquely support previous speculations that hand-selective IPS, and possibly LOTC, could be functionally distinct from tool-selective regions, despite their anatomic overlap (Striem-Amit et al., 2017). This pattern of results is unlikely to be driven by differences in ROI radius (Etzel et al., 2013) since voxel size differences were negligible between hand- and tool-selective ROIs (mean difference: IPS, 29; LOTC, 4). In fact, if category-related results were merely caused by ROI size, then significant decoding should have also been observed in the much larger LOTC-object ROI (Table 1). Alternatively, successful higher decoding in hand than in tool-selective areas might reflect that our task simply required grasping to touch the tools, rather than their utilization. That is, coding in category-selective areas might operate in an effector-dependent manner, akin to how tool-selective pMTG/LOTC codes the type of action being performed when holding a pair of tongs, but not if being performed by the hand alone (Gallivan et al., 2013). In line with this interpretation, neural representations in LOTC-hand of one-handed amputees are also known to become richer as prosthetic usage increases (Van den Heiligenberg et al., 2018), which, again, indicates that the representations in hand-selective cortex depend on effector use. An alternative, but not mutually exclusive, possibility is that only tool-use actions elicit tool-selective representations (Randerath et al., 2010) because of the cognitively taxing demands these complex actions rely on, such as retrieving knowledge about manipulation hierarchies (Buxbaum, 2017) or the laws that constrain object movement (Fischer et al., 2016). In either case, the specificity of decoding typical tool grasps in hand-selective, rather than tool- and hand-selective. cortex challenges the popular interpretation that brain activation for viewing tool images is a reflection of sensorimotor processing linked to tool manipulation (Martin et al., 1996; Grafton et al., 1997; Martin and Chao, 2001; Fang and He, 2005; Mahon et al., 2007; see also Mahon and Caramazza, 2009).

There are several differences between our study and previous research. First our univariate analysis revealed no relationship between mean activity and typicality. Previous studies have found greater univariate activation in occipitotemporal and/or frontoparietal cortex for typical relative to atypical actions when participants viewed pictures and movies or pantomimed (Johnson-Frey et al., 2003; Valyear and Culham, 2010; Yoon et al., 2012; Mizelle et al., 2013; Przybylski and Króliczak, 2017). Our results fit the claim that MVPA can reveal fine-grained effects (Kriegeskorte et al., 2006); as recently argued by Buchwald et al. (2018) when showing that pantomimed typical tool versus nontool grasps could be decoded from a range of regions including premotor and intraparietal areas. We suspect that task differences are also an important contributing factor to the general lack of univariate effects. For example, our experiment involved fewer, less varied, exemplars than in these previous picture studies. Likewise, our grasp-to-touch paradigm is simpler than studies showing greater univariate activations in the left SMG, premotor cortex, LOTC, and IPS when performing real tool-use actions (Valyear et al., 2012; Brandi et al., 2014) or haptically guided typical tool grasps (Styrkowiec et al., 2019) relative to tool/nontool control actions. Finally, in our study, grasping always involved a precision grip, whereas previous studies used power grasps, which are better suited for certain actions with some specific tools. This factor may have led to the lack of typicality decoding effects in tool-selective cortex as these areas could be sensitive to both the side of the object being grasped and the function of particular grips (Buxbaum et al., 2006). We designed our precision grasping task to investigate tool affordances while carefully equating biomechanics between actions, such that decoding typicality was unlikely to be attributed to motor-related differences. Future real-action studies manipulating the type of grasp (e.g., grasp vs use) are needed to further identify the content of information coded by visual hand-/tool-selective areas.

It is worth noting that we were unable to match the visual symmetry between object categories (our tools were asymmetric while the nontools were symmetric) because asymmetric nontool bars were perceived as tools by participants (i.e., the wider side perceived as a functional end). Nonetheless, tool-specific decoding in hand-selective cortex is unlikely to be explained by simple effects of symmetry: if effects were related to symmetry, comparable decoding effects should have been observed in symmetry-responsive regions (e.g., LOTC-object, EVC; Beck et al., 2005), particularly since they are also known to code motor-related information (Gallivan and Culham, 2015; Monaco et al., 2020).

In conclusion, parietal and occipital visual regions specialized for representing hands were found to encode information about the functional relationship between the grasping hand and a tool, implicating hand-selective cortex in motor control. These findings raise novel questions about the possibility that overlapping hand- and tool-selective regions are functionally distinct and begin to uncover which brain regions evolved to support tool use, a defining feature of our species.
